# Is there hybridization between diploid and tetraploid *Euphrasia* in a secondary contact zone?

**DOI:** 10.1002/ajb2.16100

**Published:** 2022-12-26

**Authors:** Max R. Brown, Hannes Becher, Sebastian Williams, Alex D. Twyford

**Affiliations:** ^1^ Institute of Ecology and Evolution University of Edinburgh, Ashworth Laboratories, Charlotte Auerbach Road Edinburgh EH9 3FL UK; ^2^ Wellcome Sanger Institute Wellcome Genome Campus, Hinxton Cambridge CB10 1SA UK; ^3^ Royal Botanic Garden Edinburgh, 20a Inverleith Row Edinburgh EH3 5LR UK

**Keywords:** cross‐ploidy, *Euphrasia*, genotyping by sequencing, hybridization, introgression, Orobanchaceae, secondary contact

## Abstract

**Premise:**

Strong postzygotic reproductive isolating barriers are usually expected to limit the extent of natural hybridization between species with contrasting ploidy. However, genomic sequencing has revealed previously overlooked examples of natural cross‐ploidy hybridization in some flowering plant genera, suggesting that the phenomenon may be more common than once thought. We investigated potential cross‐ploidy hybridization in British eyebrights (*Euphrasia*, Orobanchaceae), a group from which 13 putative cross‐ploidy hybrid combinations have been reported based on morphology.

**Methods:**

We analyzed a contact zone between diploid *Euphrasia rostkoviana* and tetraploid *E. arctica* in Wales. We sequenced part of the internal transcribed spacer (ITS) of nuclear ribosomal DNA and used genotyping by sequencing (GBS) to look for evidence of cross‐ploidy hybridization and introgression.

**Results:**

Common variant sites in the ITS region were fixed between diploids and tetraploids, indicating a strong barrier to hybridization. Clustering analyses of 356 single‐nucleotide polymorphisms (SNPs) generated using GBS clearly separated samples by ploidy and revealed strong genetic structure (*F*_ST_ = 0.44). However, the *F*_ST_ distribution across all SNPs was bimodal, indicating potential differential selection on loci between diploids and tetraploids. Demographic inference suggested potential gene flow, limited to around one or fewer migrants per generation.

**Conclusions:**

Our results suggest that recent cross‐ploidy hybridization is rare or absent in a site of secondary contact in *Euphrasia*. While a strong ploidy barrier prevents hybridization over ecological timescales, such hybrids may form in stable populations over evolutionary timescales, potentially allowing cross‐ploidy introgression to take place.

Natural hybridization is an important evolutionary phenomenon with wide‐ranging consequences, from extinction (Rhymer and Simberloff, 1996) to hybrid speciation (Hegarty and Hiscock, 2005). Most studies to date have investigated hybridization between diploid species, while hybridization between species that differ in their ploidy level (cross‐ploidy hybridization) has generally received less attention. While this may, in part, be due to technical challenges of inferring homological relationships between genetic markers in diploids and polyploids, there are also clear biological reasons. Contrasting ploidy levels represent a known, highly effective barrier to hybridization (Husband and Sabara, 2004). The main barriers are (1) abnormal endosperm ratios of maternal to paternal genomes at fertilization that prevent hybrid seed formation (Johnston et al., 1980) and (2) later hybrid sterility caused by irregularities in chromosome pairing at meiosis leading to aneuploid gametes (Tate et al., 2005). Both of these factors prevent initial hybrid formation and introgression between species with contrasting ploidy levels. These barriers can be overcome through unreduced gamete production in the lower‐ploidy parent, or where a triploid (or other intermediate ploidy) F_1_ hybrid is formed, either by backcrossing to one of the parental species (Ramsey and Schemske, 1998) or by whole‐genome duplication to restore fertility (Abbott and Lowe, 2004). Cross‐ploidy hybridization may be an important mechanism for maintaining genetic variation in polyploid species (although distinguishing this from recurrent polyploidization can be difficult; see Shimizu‐Inatsugi et al., 2009), exchanging adaptive alleles between species (Chapman and Abbott, 2010), and generating new polyploid cytotypes or species (Abbott and Lowe, 2004).

Examples of natural cross‐ploidy hybridization reported in the literature span many phylogenetically distinct plant lineages, including *Chrysanthemum* (Qi et al., 2021), *Dactylorhiza* (De Hert et al., 2012), *Mercurialis* (Buggs and Pannell, 2007), and *Epidendrum* (Pinheiro et al., 2010). Where hybridization is particularly common, hybrid swarms can develop, as seen in co‐occurring diploid and tetraploid species of *Cochlearia* (Fearn, 1977). Cross‐ploidy hybridization in the genera *Senecio* and *Mimulus* have led to the formation of three hybrid species endemic to Britain (Abbott and Lowe, 2004; Vallejo‐Marin, 2012). Two of these hybrid species have resulted from whole‐genome duplication of initial triploid F_1_ hybrids (*Senecio cambrensis* and *Mimulus peregrinus*), while the other species was created through introgression to the tetraploid parent (*S. eboracensis*). Although not exhaustive, these examples highlight that cross‐ploidy hybridization often involves diploid and tetraploid species, which are the cytotypes that most commonly co‐occur (Kolar et al., 2017), and that, when introgression happens, it is usually in the direction of the higher‐ploidy parental species. Here, the higher likelihood of unreduced gamete formation by the lower‐ploidy parent may allow it to form fertile lineages with the higher‐ploidy parent (Stebbins, 1971; Baduel et al., 2018). Moreover, the likelihood and extent of introgression is also affected by the mode of parental ploidy and the relationship between diploids and tetraploids. For example, cross‐ploidy hybridization is more likely if it involves allopolyploids, which may exhibit preferential pairing between chromosomes homeologous between the diploid and allotetraploid and, therefore, regular meiosis (M. R. Brown et al., unpublished data).

*Euphrasia* (Orobanchaceae) is a large temperate genus of hemiparasitic plants, with around 263 species worldwide (D. Nickrent, personal communication). There are 21 *Euphrasia* species in Britain and Ireland, representing a taxonomically complex group characterized by recent postglacial divergence (Wang et al., 2018), phenotypic plasticity (Brown et al., 2020), and rampant hybridization (Metherell and Rumsey, 2018). British and Irish *Euphrasia*, all annuals, consist of five predominantly outcrossing diploid species and 16 selfing or mixed‐mating tetraploid species (French et al., 2005). Genomic analysis has revealed an allopolyploid origin of the tetraploids, one progenitor of which is likely a relative of a British diploid, while the other, divergent progenitor remains unknown (Becher et al., 2020). The high degree of tetraploid subgenome divergence (~5%; Becher et al., 2020) has benefits for genomic analyses, increasing confidence in the assignment of short reads to the appropriate subgenome. British tetraploids are thought to have a common origin in mainland Europe and to have subsequently colonized the British Isles (Garrett et al., 2022).

Out of 71 hybrid *Euphrasia* combinations reported in the British flora based on morphology, 13 are reported to be diploid‐tetraploid hybrids (Stace et al., 2015; Figure 1A). Unusually for cross‐ploidy hybrids, these are purported to be diploids, derived from triploid F_1_ hybrids (Yeo, 1956), with the putative cross‐ploidy hybrid *E. anglica* × *E. nemorosa* confirmed as diploid by flow cytometry (Becher et al., 2021). In this proposed cross‐ploidy hybridization scenario, the subgenome of the tetraploid species homologous to extant diploids can successfully pair and recombine, while the divergent tetraploid subgenome is rapidly lost in meiosis (Figure 1B). This may allow introgression into the diploid species through backcrossing to the diploid parent.

**Figure 1:**
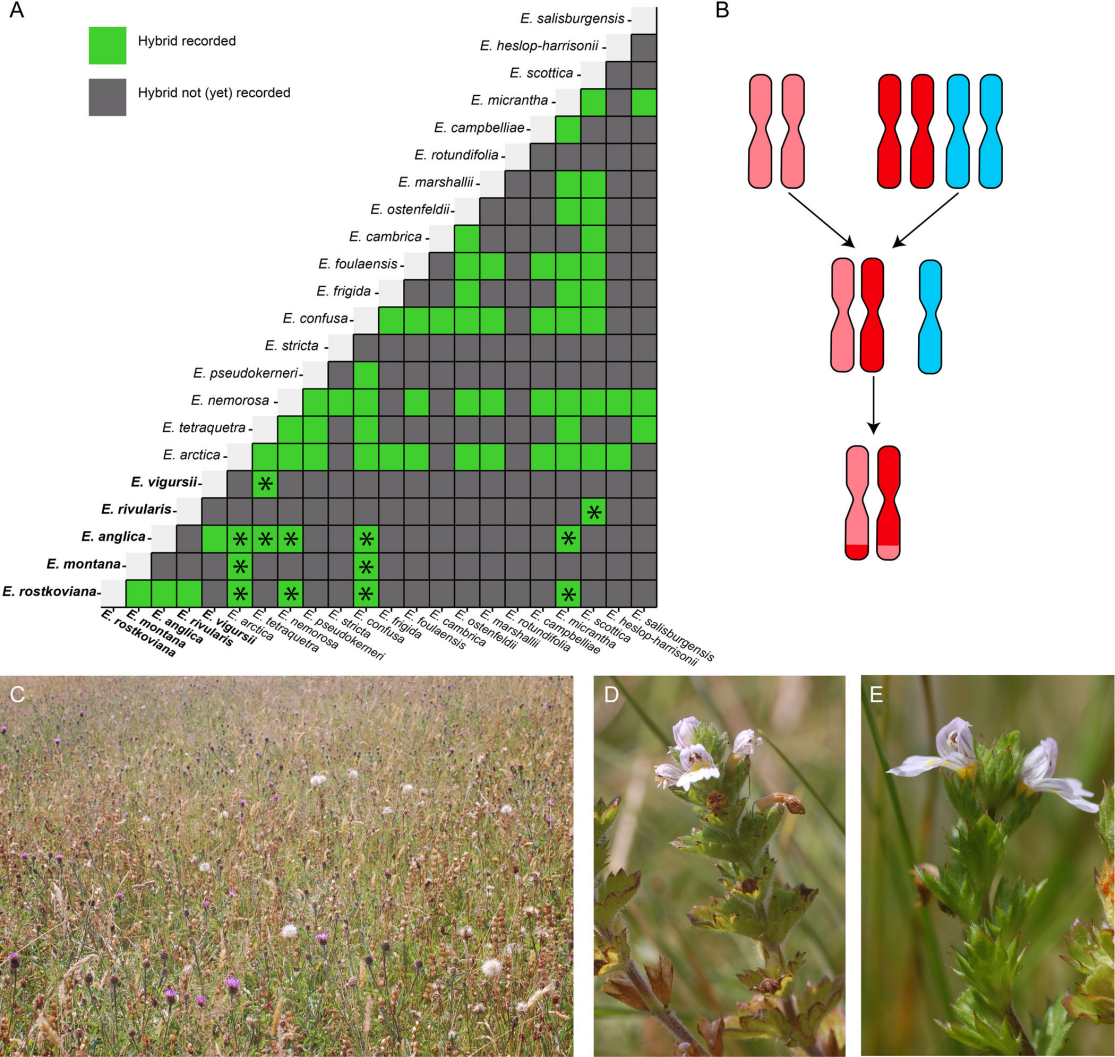
Crossing relationships in British and Irish *Euphrasia*. (A) Putative hybrids based on morphology recorded between all 21 confirmed native *Euphrasia* species in addition to *E. stricta*, which is of questionable status. Diploid species are in bold, and cross‐ploidy hybrids are indicated with an asterisk. Data are from Metherell and Rumsey (2018). (B) Hypothesized genomic similarity and chromosome pairing between diploid and tetraploid species. Diploid chromosomes (pink) are hypothesized to pair with syntenous chromosomes in the tetraploid (red) and not with chromosomes from the divergent subgenome (blue), allowing the formation of a stable diploid hybrid and introgression into the diploid species. (C) The managed hay meadow at Cae Trawscoed, where diploid and tetraploid *Euphrasia* species grow in sympatry. (D) Diploid *Euphrasia rostkoviana*. (E) Tetraploid *Euphrasia arctica*. An interactive version of the plot in A is available at bit.ly/Eupcrossinggrid.

We investigated the potential for hybridization and introgression in a contact zone between diploid *E. rostkoviana* and tetraploid *E. arctica* (Figure 1). Both of these species are widespread and are reported to hybridize locally at a number of sites across Britain, based on morphological observations (Stace et al., 2015). We first used Sanger sequencing of a locus that shows diagnostic differences between diploid and tetraploid species (Wang et al., 2018) to see if there is any clear evidence of hybridization. We then used genotyping by sequencing (GBS) to generate single‐nucleotide polymorphism (SNP) markers and test for evidence of potentially cryptic introgression. We also used demographic modeling to investigate the range of scenarios of historical hybridization that may explain the observed genetic structure. Given that reproductive barriers between *Euphrasia* species are known to be exceptionally weak, and that cross‐ploidy hybridization is increasingly confirmed by genomic data in other plant groups, we predicted the presence of cross‐ploidy hybrids and evidence of introgression.

## MATERIALS AND METHODS

### Population sampling and DNA extraction

Sampling took place in July 2017 in a managed hay meadow at Cae Trawscoed in the National Botanical Garden Wales (51.8447, −4.14531), where there is a mixed population of diploid *E. rostkoviana* and tetraploid *E. arctica*. These species can be readily separated based on the presence or absence of long‐stalked flexuous glandular hairs, which are present in all British diploid species but absent in all British tetraploids, as well as a suite of other floral, branching, leaf, and capsule characters (Metherell and Rumsey, 2018). Putative early‐generation hybrids between species show a low density of long glandular hairs and are intermediate for other traits (Stace et al., 2015); however, no clearly morphologically intermediate individuals were encountered while sampling at this site. Both species are characterized by large corollas and are likely to be outcrossers or mixed‐maters (French et al., 2005; Metherell and Rumsey, 2018). Species identities were confirmed by the *Euphrasia* referee Chris Metherell and herbarium samples are lodged at Edinburgh (E).

The species are broadly intermixed at the site, with *E. rostkoviana* present among taller vegetation and *E. arctica* more dominant in shorter vegetation. Both species are abundant and present in their thousands. A total of 95 individuals were sampled from the mixed population, 45 being identified by morphology as diploid and 50 as tetraploid. Plants were sampled from a transect of ~12 m where plants were intermixed. Specimens were collected into silica gel prior to DNA extraction using the DNeasy Plant Mini kit (Qiagen, Germantown, Maryland, USA), following the manufacturer's protocols.

### Sanger sequencing and sequence analysis

We sequenced 70 *Euphrasia* individuals for part of the internal transcribed spacer of nuclear ribosomal DNA (ITS) as this locus demonstrates diagnostic differences between diploid and tetraploid species (Wang et al., 2018). We also performed a preliminary survey of plastid variation by sequencing the variable trnL spacer region (rpL32‐trnL^UAG^) in a test panel of four diploid and four tetraploid individuals. All PCRs were performed in 25 μL reactions; DNA amplification protocols and conditions for PCRs are given in Appendix S1. PCR products were visualized on a 1% agarose gel, cleaned with ExoSAP‐IT (Life Technologies, Darmstadt, Germany) using standard protocols, and submitted to Edinburgh Genomics for sequencing reactions using the BigDye Terminator Cycle Sequencing Kit version 3.1 (Life Technologies, Carlsbad, California, USA) and Sanger Sequencing on an ABI 3730. ITS PCR products were sequenced in the forward direction only (with the ITS4 primer), while the trnL spacer was sequenced in both directions.

Sequence chromatograms were trimmed for low‐quality bases and subsequently aligned in Geneious version 9.0.5. Eight ITS sequences were excluded due to poor sequence quality. The 558 bp trnL spacer alignment of eight sequences showed no variable sites and was therefore not analyzed further. The final ITS alignment included 62 individuals and was 658 bp in length with 58 variable sites in total. *E. transmorrisonensis* (diploid; from Taiwan), was added as an outgroup to root the ITS phylogeny, with the sequence downloaded from NCBI (GenBank accession no. AY165615). We constructed a maximum likelihood phylogeny in IQ‐TREE version 1.5.5 (Nguyen et al., 2015), using ModelFinder to find the most suitable substitution model (using the TESTNEWMERGE model flag) with 1000 ultra‐fast bootstraps (Hoang et al., 2018). The resulting newick file was visualized in ggtree version 2.1.2 (Yu et al., 2017). To further characterize sequence variation, the alignment was read into R version 3.6.1 using the function read.dna() from the package ape version 5.4 (Paradis and Schliep, 2019) and converted to a genind object with adegenet version 2.1.2 (Jombart, 2008). We tested the partitioning of genetic variation between ploidy levels using analysis of molecular variance (AMOVA) implemented in poppr version 2.8.4 (Kamvar et al., 2014) using function poppr.amova().

### GBS and SNP discovery

GBS library preparation was performed following the protocol of Elshire et al. (2011) using the frequent cutting enzyme ApeKI. Libraries were pooled and cleaned using AMPure beads before PCR amplification and sequencing. The pooled library of 95 DNA samples plus a water control was submitted to the University of Oregon for 100 bp single‐end sequencing on the Illumina HiSeq. 4000, generating 17,397,350 sequence reads. We then used the TASSEL 5 pipeline version 2 to discover SNPs from each uniquely barcoded individual (see Appendix S2) using default settings, except that k‐mer length was increased to 75 to maximize the recovery of variable sites (Glaubitz et al., 2014). Sequence tags were aligned to the draft *E. arctica* genome (Becher et al., 2020) using BWA version 0.7.17 (Li and Durbin, 2009). All genome scaffolds smaller than 1000 bp were merged into a single scaffold to reduce computational time. The VCF file was filtered for variants with >50% missing data and individuals with >75% missing data using vcftools version 0.1.16 (Danecek et al., 2011).

We focused our analyses of genetic structure, hybridization, and introgression on genomic regions that are homologous between diploids and tetraploids and likely to belong to the subgenome shared between ploidy levels (i.e., pink or red chromosomes in Figure 1B). As such, variants were excluded if they were not located on the conserved set of scaffolds identified in genome‐wide sequence comparisons of diploid and tetraploid *Euphrasia* by Becher et al. (2020). The conserved scaffolds comprise 46 Mbp (*n* = 3454) of sequence that represents part of the conserved subgenome. Therefore, sites are excluded if they are within the tetraploid restricted subgenome, where gene exchange with diploids is unlikely, or in scaffolds where subgenome assignment is uncertain. A minor allele frequency filter was applied to remove invariant sites using vcftools. To remove variants in tight linkage, only one variant was kept per scaffold by filtering with PLINK version 1.9 (Purcell et al., 2007). This left variable unlinked sites from the conserved subgenome for subsequent analyses.

### Identifying hybridization between ploidy levels

To investigate hybridization between the two *Euphrasia* species in the contact zone, we first conducted a principal component analysis (PCA) of the GBS data. The VCF was loaded into R using the package vcfR version 1.1 (Knaus and Grunwald, 2017) and the PCA was carried out using the adegenet (Jombart, 2008) function dudi.pca(), where missing values were substituted by the mean allele frequencies. The PCA was visualized using ggplot2 version 3.2.1 (Wickham, 2009). Second, we performed a Bayesian admixture analysis in Structure version 2.3.4 (Pritchard et al., 2000) using the same GBS data set. We chose STRUCTURE because it is considered the most robust clustering method for studies investigating mixed ploidy levels (Stift et al., 2019). The VCF was converted to STRUCTURE format using PGDSpider version 2.1.1.5 (Lischer and Excoffier, 2012). We set the *K*‐value to 2 to estimate genomic proportions assigned to the two divergent species, and the run was set with a burn‐in of 100,000 for 1 million iterations using the “admixture” option. The 90% probability intervals were stored using the ANCESTPINT option; samples with probability intervals overlapping 0 or 1 were considered nonhybrids. The *Q*‐matrix and probability intervals were extracted from the output and plotted using a custom R script (https://github.com/Euphrasiologist/StructuRe). Third, we explicitly attempted to identify hybrid individuals using NewHybrids version 1.1 (Anderson and Thompson, 2002), which classifies individuals into one of six potential categories (parent A, parent B, F1, F2, backcross (BC)1 to parent A, BC1 to parent B) based on their SNP genotypes. The model was run with a burn‐in of 100,000 iterations followed by a run length of 100,000 sweeps.

### Quantification of genetic variability within and between ploidy levels

To understand population structure in the contact zone, we computed several population genetic statistics on the GBS data and used AMOVA (as above) to detect SNPs that may have introgressed. Weir and Cockerham's estimator of *F*_ST_ was calculated for each SNP using vcftools (Danecek et al., 2011) and visualized in ggplot2 (Wickham, 2009). The average *F*_ST_ across all SNPs was reported as the global *F*_ST_. An AMOVA was run for both the ITS and GBS data sets using the function poppr.amova() from the R package poppr (Kamvar et al., 2014), which was used to understand the partitioning of genetic variability both within and between ploidy levels. *P*‐values were then derived from the output using the randtest() function from ade4 version 1.7‐15 (Bougeard and Dray, 2018), randomly permuting sample matrices 9999 times.

### Demographic inference with δaδi

We carried out demographic modeling using the package δaδi to test the best‐fitting model of historical hybridization for the GBS data (Gutenkunst et al., 2009). We used δaδI to understand the general demographic history of secondary contact between species—considering population sizes and migration rates as a function of time—without incorporating selection on single sites in our relatively sparse SNP data. We handled missing data by scaling down the joint site‐frequency spectra to 24 (haploid) genomes per species. We implemented four models, each involving one ancestral population splitting into two subpopulations corresponding to diploids and tetraploids, which could differ in their population sizes. Model parameters corresponding to the diploid and tetraploid subpopulations are denoted by subscript D and T. The models differed by the amount of gene flow allowed between the subpopulations: (1) constant gene flow, with five parameters (two population sizes, N_eD_ and N_eT_; two migration rates, M_DT_ and M_TD_; and the time of the population split, T_0_); (2) historical gene flow only, with six parameters (two population sizes; two migration rates; time when gene flow ceased, T_1_; and the difference between T_1_ and the time of the split, denoted T_0_); (3) secondary contact, with the same parameters as in 2, but with gene flow in T_0_ and not in T_1_; or (4) no gene flow, with three parameters (two population sizes and T_0_). In the absence of a mutation rate estimate, we set the ancestral effective population size N_e_ to 1. The effective sizes of the current populations, N_eD_ and N_eT_, are given as proportions of the ancestral N_e_. We fixed the inbreeding coefficient *F* at 0.75 and 0.81 for *E. arctica* and *E. rostkoviana*, respectively, according to preliminary δaδI estimates from the site‐frequency spectra of our data sets. While these inbreeding values are useful for the demographic models, they will be overestimates in relation to the actual *F*, due to the undercalling of heterozygous sites in GBS data. To assess the uncertainty of the model fitting, we used 99 individual subsamplings of our data. Because we found the model‐fitting results to depend strongly on the initial conditions, we ran 99 replicates with randomly perturbed starting values for each model and a down‐sampled data set resulting in 39,204 optimizations. From each set of 99 replicates, we selected the one with the best log likelihood. In order to compare these nested models with different numbers of parameters, we computed the Akaike Information Criterion (AIC) of each fit, and plotted the results with matplotlib to allow for comparisons (Hunter, 2007).

## RESULTS

### Patterns of cross‐ploidy genetic structure observed with ITS

**Figure 2:**
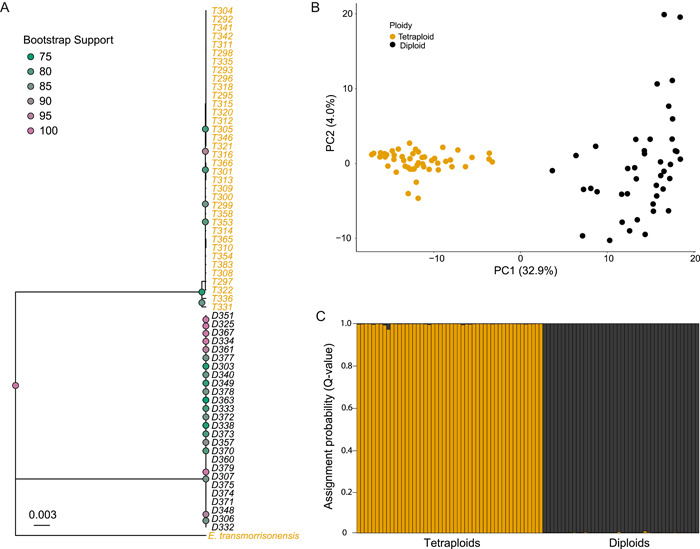
ITS and GBS analyses suggest strong barriers to gene flow between diploid (black) and tetraploid (orange) species in the genus *Euphrasia*. (A) Maximum likelihood phylogenetic tree of ITS sequences generated with IQ‐TREE, with *E. transmorrisonensis* as an outgroup. Bootstrap support is visualized as color‐scaled points at each node, with only values >75 shown. (B) Principal component analysis of 356 unlinked SNPs loci for 92 *Euphrasia* individuals. The first two principal components are plotted against each other. (C) Structure analysis on the SNP data with *K* = 2.

The final alignment of the ITS sequence data included 26 diploid and 36 tetraploid individuals. The chromatograms revealed no evidence of sequence additivity or double peaks, which might have indicated hybrid individuals or retained duplicate copies in the tetraploids. The maximum likelihood phylogenetic tree showed two distinct and highly supported clades (Figure 2A). There were 58 SNPs in the alignment fixed between ploidy groups, while most other SNPs were rare singletons. Accordingly, an analysis of molecular variance (AMOVA) showed that 99.5% of the ITS variation in the samples was explained by ploidy (*p* < 0.001; Appendix S3).

### Patterns of genetic structure from GBS data

Analyses of 356 unlinked SNPs representing putatively disomically inherited scaffolds shared between diploids and tetraploids (see above) corroborated the findings from the ITS sequences, with the multilocus SNP data matching the morphological assignment for all individuals. Principal component analysis (PCA) showed that the first principal component explained 32.9% of the genomic variation and clearly separated individuals by ploidy, with two separate clusters and no evidence of intermediate genotypes (Figure 2B). The same pattern was also present in the Structure analysis with a *K* value of 2, which reported all *Q*‐values per individual to be >0.98 (Figure 2C), assigning individuals to clusters corresponding to species. The probability interval of each *Q*‐value overlapped either 0 or 1, indicating “pure” parental genotypes. The NewHybrids analysis assigned each individual as belonging 100% to one of the parental species, with no evidence of F_1_ hybrids or backcross individuals (results not shown). An AMOVA on the GBS data showed that 78.4% of genomic variation was explained by ploidy (*p* < 0.001; Appendix S3). The remainder of the genomic variation (21.6%) was due to differences within ploidy level.

### Detecting hybridization across the genome

**Figure 3:**
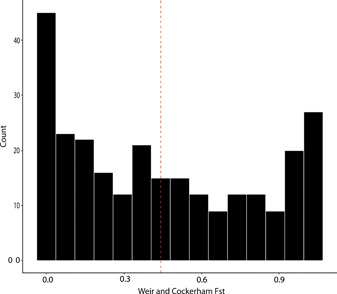
The distribution of *F*_ST_ for each of 356 SNPs in the GBS data set across all 92 *Euphrasia* individuals between the two ploidy levels. The red dashed line indicates mean global *F*_ST_ (0.44).

We calculated population genetic parameters from the GBS data to further investigate potential hybridization between the two species differing in ploidy level. The global *F*_ST_ between species was high at 0.44, indicating that diploid *E. rostkoviana* and tetraploid *E. arctica* are highly differentiated across the genome. The distribution of *F*_ST_, however, showed a bimodal distribution with a high count of SNPs that were either mostly shared or private (Figure 3).

### Model selection suggests low levels of gene flow

**Figure 4:**
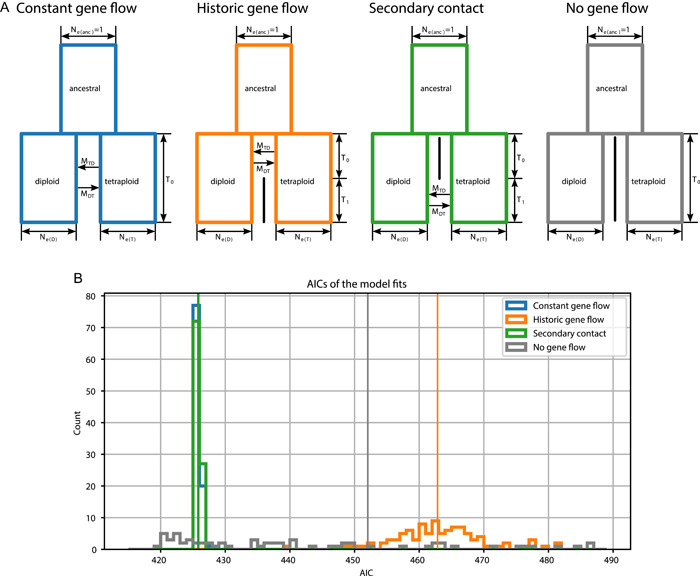
Demographic model fitting for 356 SNPs generated via GBS for diploid and tetraploid *Euphrasia* species. (A) Schematics of the demographic models and their parameters. N_e_, the effective population size, is scaled to be 1 in the ancestral population. N_eD_ and N_eT_ indicate the ratios of the present‐day effective population sizes of the diploid and tetraploid populations, respectively, in relation to the ancestral population. T is the number of generations to coalescence, which is subdivided into two episodes in the models, with ancestral gene flow and secondary contact. M is the number of migrants per generation, with subscripts indicating the direction of gene flow. (B) The distributions of AIC values for model fits to 99 subsampled data sets. The models with constant gene flow and secondary contact are not distinguishable. Vertical lines indicate the distribution medians.

The best‐supported demographic models fitted with δaδI were those with constant gene flow or with secondary contact (Figure 4; Appendix S4). These models consistently scored low AIC values. The difference in AIC between the best model (constant gene flow, median AIC 425.77) and the runner‐up (secondary contact, median AIC 425.89) was not significant (*t* = −1.38, *p* = 0.17). The other two models had significantly higher median AICs than the best model (historical gene flow: 462.83, *t* = −7.29, *p* < 0.001; no gene flow: 452.05, *t* = −52.31, *p* < 0.001). However, for some realizations of the data resampling, the model with no gene flow scored the best AIC, coinciding with generally low estimates of the age of the population divergence time (T_0_ tends to be lower without gene flow than in alternative models; see Appendix S1).

The levels of gene flow fitted in our models tended to be low, with *M*, the number of migrants per generation, ≤1. The mean values over all replicates for the constant gene flow model were 0.3 (diploid → tetraploid, SD = 0.09) and 0.4 (tetraploid → diploid, SD = 0.13). For the model with secondary contact the migration rates were fitted to be 1.3 (diploid → tetraploid, SD = 1.02) and 0.3 (tetraploid → diploid, SD = 0.09). In all models, the effective population size tended to be slightly higher in the diploids than in the tetraploids (Appendix S1, left‐hand panels). While our data suggest the presence of low levels of gene flow, the similar AIC values we obtained under different models show how different demographic scenarios may produce similar genetic patterns.

## DISCUSSION

We investigated secondary contact between diploid *E. rostkoviana* and tetraploid *E. arctica* in a managed meadow in South Wales. The prevalence of hybridization between *Euphrasia* species of the same ploidy level (Stace et al., 2015; Metherell and Rumsey, 2018; Zlonis and Gross, 2018; Becher et al., 2020) and observations of 13 putative diploid‐tetraploid hybrids based on morphology led us to expect the presence of cross‐ploidy hybrids. However, neither phylogenetic analyses using ITS—which is known to have diagnostic differences between ploidy levels—nor population structure analyses using hundreds of SNP markers generated with GBS showed any evidence of recent hybridization between the species. While this may seem surprising, to date only a single wild triploid *Euphrasia* hybrid individual has been reported (Yeo, 1956), and previous attempts to artificially generate such hybrids have been unsuccessful (Yeo, 1968). One possible explanation may be that initial hybrid formation is rare and remains undetectable over short (ecological) timescales, but that rare hybrids may form, establish, and backcross over longer (evolutionary) timescales. This is a common outcome in many plant groups; for example, in Louisiana *Iris* hybrid zones, F_1_ hybrids form at a low rate («1%), with only a single wild collected plant out of 768 considered an F_1_ based on its genotype (Johnston et al., 2001). As such, we cannot exclude the possibility that rare early‐generation hybrids were overlooked in our sample of individuals used for GBS. Future studies will sequence putative diploid‐tetraploid hybrid individuals identified based on morphology, to confirm their origins, ploidy, and genomic composition.

Although our conventional population genetic analyses did not indicate recent gene flow between the two *Euphrasia* species, demographic modeling using δaδI, and inspection of the distribution of *F*_ST_ values across all SNPs, indicated that there may be limited gene flow, compatible with low amounts of introgression over longer timescales. We also note that our limited plastid sequencing revealed shared plastid haplotypes between these divergent species, which may represent plastid capture, as has been proposed previously (Wang et al., 2018). Model estimates with δaδI gave low estimates of bidirectional gene flow, which is known to occur in other cases where cross‐ploidy hybridization occurs (Bleeker, 2003). In particular, the constant gene flow model gave a higher estimate of tetraploid to diploid gene flow, which may support the hypothesis for (directional) introgression in *Euphrasia* made >60 years ago (Yeo, 1956). However, our overall power to detect the directionality of introgression is limited and the alternative model of diploid‐tetraploid introgression receives some support. This lack of power is likely due to the number of SNPs, as we focused on the few hundred sites confidently known to be present in the subgenome conserved between diploids and tetraploids. The fact that the signature of gene flow was weak may also be due to introgression being restricted to small genome regions, many of which will be undersampled with our sparse SNP markers. Moreover, selection may be operating differentially between genomic regions, which may explain the bimodal *F*_ST_ distribution seen in our data set (Whitlock, 2008), and this could be incorporated in future modeling with more extensive genomic data.

Our results are consistent with some cross‐ploidy hybrid systems in which hybridization is very rare or hybrids are strongly selected against. For example, in diploid *Centaurea pseudophrygia* and tetraploid *C. jacea*, only targeted sampling was able to reveal cross‐ploidy hybrids, which were otherwise not found by random sampling in 12 different contact zones (Koutecky et al., 2011). In a contact zone of diploid *Senecio madagascariensis* and tetraploid *S. pinnatifolius*, no hybrids have been detected in the field; however, genetic analysis of the seeds revealed hybrid genotypes (Prentis et al., 2007). It is possible in the *Euphrasia* contact zone that hybrid seed is being formed but either it does not germinate or hybrid seedlings do not survive to maturity, a possibility that could be tested by screening the ploidy of many wild‐collected seedlings with flow cytometry. Our results stand in strong contrast to those found in *Dactylorhiza*, where triploid F_1_ cross‐ploidy hybrids and backcrossed individuals are found frequently (De Hert et al., 2012; Balao et al., 2017). The mechanisms underlying these differences in the frequency of cross‐ploidy hybrids in divergent species are yet to be fully established, and this is likely to be an area for fruitful future research.

## AUTHOR CONTRIBUTIONS

A.D.T. and M.R.B. designed the research. M.R.B. and S.W. generated sequence data. M.R.B. and H.B. analyzed the data. A.D.T. and M.R.B. drafted the manuscript. All authors contributed to and agreed on the final manuscript.

## Supporting information


**Appendix S1**. Primers and PCR conditions for Sanger sequencing (adapted from Wang et al., 2018).

**Appendix S2**. Keyfile for demultiplexing Illumina short reads for the GBS data set.

**Appendix S3**. Hierarchical analysis of molecular variance (AMOVA) for ITS and 356 SNPs generated via GBS for diploid *Euphrasia rostkoviana* and tetraploid *E. arctica*.

**Appendix S4**. Distributions of parameters fitted to the models with constant gene flow, with secondary contact, and without gene flow.

## Data Availability

Raw Illumina sequence reads are available on the SRA as part of BioProject PRJNA892273, with the keyfile required for demultiplixing included in Online Supporting Information. The GBS and Sanger sequence alignments are available on the Dryad Digital Repository: https://doi.org/10.5061/DRYAD.3J9KD51NR (Brown et al., 2022). The scripts are available on github, including those for calling SNPs from the GBS data (https://github.com/Euphrasiologist/GBS_V2_Tassel_5), for STRUCTURE analyses (https://github.com/Euphrasiologist/StructuRe) and for demographic model inference (https://github.com/hannesbecher/Euphrasia-demographic-modeling).
