# Supplementary material for: Is there hybridization between diploid and tetraploid Euphrasia in a secondary contact zone?
Source: Am J Bot. 2022 Dec 26;110(1):e16100. doi: 10.1002/ajb2.16100 (PMC10107515; doi:10.1002/ajb2.16100)
Supplement: Supplementary file 1 — Appendix S1. Primers and PCR conditions for Sanger sequencing (adapted from Wang et al., 2018). [file AJB2-110-0-s001.docx]

Appendix S1. Primers and PCR conditions for Sanger sequencing. Adapted from Wang et al. (2018).

| Genome | Primer | Orientation | Sequence (5’-3’) | Reagents (1 reaction) | PCR conditions |
| --- | --- | --- | --- | --- | --- |
| Plastid | rpL32-F | Forward | CAGTTCCAAAAAAACGTACTTC | 12.5μM Taq 2X Master Mix, 0.5μL Bovine Serum Albumin (BSA), 0.5μL forward and reverse primers at 10μM, 10.5μL water, 1μL sample DNA | 5 min at 94 ^o^C, 35× (30 s at 94 ^o^C, 45 s at 50 ^o^C, 40 s at 72 ^o^C), 5 min at 72 ^o^C |
|  | trnL^UAG^ | Reverse | CTGCTTCCTAAGAGCAGCGT |  |  |
| Nuclear ribosomal | ITS4 | Forward | TCCTCCGCTTATTGATATGC | 12.5μM Taq 2X Master Mix, 0.5μL BSA, 0.5μL forward and reverse primers at 10μM, 10.5μL water, 1μL sample DNA | 5min at 94°C, 30 x (30s at 94°C, 30s at 54°C, 2min at 72°C), 10 min at 72°C. |
|  | ITS5 | Reverse | GGAAGTAAAAGTCGTAACAAGG |  |  |
