# Supplementary material for: Is there hybridization between diploid and tetraploid Euphrasia in a secondary contact zone?
Source: Am J Bot. 2022 Dec 26;110(1):e16100. doi: 10.1002/ajb2.16100 (PMC10107515; doi:10.1002/ajb2.16100)
Supplement: Supplementary file 2 — Appendix S2. Keyfile for demultiplexing Illumina short reads for the GBS data set. [file AJB2-110-0-s004.docx]

Appendix S2. Keyfile for demultiplexing Illumina short reads for the GBS dataset. Table in the standard format required for Tassel (Glaubitz et al. 2014). See the Tassel handbook for information on the column headers.

| Flowcell | Lane | Barcode | FullSampleName | PlateName | Row | Col | LibraryPrepID | LibraryPlateID | Enzyme |  | Genus | Species | Pedigree |
| --- | --- | --- | --- | --- | --- | --- | --- | --- | --- | --- | --- | --- | --- |
| HGHVLBBXY | 3 | AGGC | T291 | Euphrasia1 | A | 1 | 100000100 | 160000001 | ApeKI | A1 | Euphrasia | spp | wildcollected |
| HGHVLBBXY | 3 | GATT | T292 | Euphrasia1 | A | 2 | 100000101 | 160000001 | ApeKI | A2 | Euphrasia | spp | wildcollected |
| HGHVLBBXY | 3 | CGAA | T293 | Euphrasia1 | A | 3 | 100000102 | 160000001 | ApeKI | A3 | Euphrasia | spp | wildcollected |
| HGHVLBBXY | 3 | GTCC | T294 | Euphrasia1 | A | 4 | 100000103 | 160000001 | ApeKI | A4 | Euphrasia | spp | wildcollected |
| HGHVLBBXY | 3 | ACAT | T295 | Euphrasia1 | A | 5 | 100000104 | 160000001 | ApeKI | A5 | Euphrasia | spp | wildcollected |
| HGHVLBBXY | 3 | GCGA | T296 | Euphrasia1 | A | 6 | 100000105 | 160000001 | ApeKI | A6 | Euphrasia | spp | wildcollected |
| HGHVLBBXY | 3 | TGCT | T297 | Euphrasia1 | A | 7 | 100000106 | 160000001 | ApeKI | A7 | Euphrasia | spp | wildcollected |
| HGHVLBBXY | 3 | CCTC | WATER1 | Euphrasia1 | A | 8 | 100000107 | 160000001 | ApeKI | A8 | Euphrasia | spp | wildcollected |
| HGHVLBBXY | 3 | AACA | T298 | Euphrasia1 | A | 9 | 100000108 | 160000001 | ApeKI | A9 | Euphrasia | spp | wildcollected |
| HGHVLBBXY | 3 | CTGT | T299 | Euphrasia1 | A | 10 | 100000109 | 160000001 | ApeKI | A10 | Euphrasia | spp | wildcollected |
| HGHVLBBXY | 3 | TAAC | T300 | Euphrasia1 | A | 11 | 100000110 | 160000001 | ApeKI | A11 | Euphrasia | spp | wildcollected |
| HGHVLBBXY | 3 | CTCGA | D301 | Euphrasia1 | A | 12 | 100000111 | 160000001 | ApeKI | A12 | Euphrasia | spp | wildcollected |
| HGHVLBBXY | 3 | ACCGT | D302 | Euphrasia1 | B | 1 | 100000112 | 160000001 | ApeKI | B1 | Euphrasia | spp | wildcollected |
| HGHVLBBXY | 3 | CGTCA | D303 | Euphrasia1 | B | 2 | 100000113 | 160000001 | ApeKI | B2 | Euphrasia | spp | wildcollected |
| HGHVLBBXY | 3 | TCGCA | T304 | Euphrasia1 | B | 3 | 100000114 | 160000001 | ApeKI | B3 | Euphrasia | spp | wildcollected |
| HGHVLBBXY | 3 | CGCAT | T305 | Euphrasia1 | B | 4 | 100000115 | 160000001 | ApeKI | B4 | Euphrasia | spp | wildcollected |
| HGHVLBBXY | 3 | GCACT | T306 | Euphrasia1 | B | 5 | 100000116 | 160000001 | ApeKI | B5 | Euphrasia | spp | wildcollected |
| HGHVLBBXY | 3 | AGCTA | D307 | Euphrasia1 | B | 6 | 100000117 | 160000001 | ApeKI | B6 | Euphrasia | spp | wildcollected |
| HGHVLBBXY | 3 | CAGCT | T308 | Euphrasia1 | B | 7 | 100000118 | 160000001 | ApeKI | B7 | Euphrasia | spp | wildcollected |
| HGHVLBBXY | 3 | TCAGC | T309 | Euphrasia1 | B | 8 | 100000119 | 160000001 | ApeKI | B8 | Euphrasia | spp | wildcollected |
| HGHVLBBXY | 3 | CATGC | T310 | Euphrasia1 | B | 9 | 100000120 | 160000001 | ApeKI | B9 | Euphrasia | spp | wildcollected |
| HGHVLBBXY | 3 | GCTAC | T311 | Euphrasia1 | B | 10 | 100000121 | 160000001 | ApeKI | B10 | Euphrasia | spp | wildcollected |
| HGHVLBBXY | 3 | CTGAC | D312 | Euphrasia1 | B | 11 | 100000122 | 160000001 | ApeKI | B11 | Euphrasia | spp | wildcollected |
| HGHVLBBXY | 3 | GACTC | T313 | Euphrasia1 | B | 12 | 100000123 | 160000001 | ApeKI | B12 | Euphrasia | spp | wildcollected |
| HGHVLBBXY | 3 | ACGTC | T314 | Euphrasia1 | C | 1 | 100000124 | 160000001 | ApeKI | C1 | Euphrasia | spp | wildcollected |
| HGHVLBBXY | 3 | CGATC | T315 | Euphrasia1 | C | 2 | 100000125 | 160000001 | ApeKI | C2 | Euphrasia | spp | wildcollected |
| HGHVLBBXY | 3 | ATGCGT | T316 | Euphrasia1 | C | 3 | 100000126 | 160000001 | ApeKI | C3 | Euphrasia | spp | wildcollected |
| HGHVLBBXY | 3 | TCCAGT | T317 | Euphrasia1 | C | 4 | 100000127 | 160000001 | ApeKI | C4 | Euphrasia | spp | wildcollected |
| HGHVLBBXY | 3 | GCTTGA | T318 | Euphrasia1 | C | 5 | 100000128 | 160000001 | ApeKI | C5 | Euphrasia | spp | wildcollected |
| HGHVLBBXY | 3 | TCCGTA | D319 | Euphrasia1 | C | 6 | 100000129 | 160000001 | ApeKI | C6 | Euphrasia | spp | wildcollected |
| HGHVLBBXY | 3 | TCGGAT | T320 | Euphrasia1 | C | 7 | 100000130 | 160000001 | ApeKI | C7 | Euphrasia | spp | wildcollected |
| HGHVLBBXY | 3 | GTCTTA | T321 | Euphrasia1 | C | 8 | 100000131 | 160000001 | ApeKI | C8 | Euphrasia | spp | wildcollected |
| HGHVLBBXY | 3 | CGGAGT | T322 | Euphrasia1 | C | 9 | 100000132 | 160000001 | ApeKI | C9 | Euphrasia | spp | wildcollected |
| HGHVLBBXY | 3 | CGTCTA | T323 | Euphrasia1 | C | 10 | 100000133 | 160000001 | ApeKI | C10 | Euphrasia | spp | wildcollected |
| HGHVLBBXY | 3 | CACGTT | T324 | Euphrasia1 | C | 11 | 100000134 | 160000001 | ApeKI | C11 | Euphrasia | spp | wildcollected |
| HGHVLBBXY | 3 | GTTAGC | D325 | Euphrasia1 | C | 12 | 100000135 | 160000001 | ApeKI | C12 | Euphrasia | spp | wildcollected |
| HGHVLBBXY | 3 | AGCATT | T326 | Euphrasia1 | D | 1 | 100000136 | 160000001 | ApeKI | D1 | Euphrasia | spp | wildcollected |
| HGHVLBBXY | 3 | CTCCGA | T327 | Euphrasia1 | D | 2 | 100000137 | 160000001 | ApeKI | D2 | Euphrasia | spp | wildcollected |
| HGHVLBBXY | 3 | TTGGCA | D328 | Euphrasia1 | D | 3 | 100000138 | 160000001 | ApeKI | D3 | Euphrasia | spp | wildcollected |
| HGHVLBBXY | 3 | CCACGT | D329 | Euphrasia1 | D | 4 | 100000139 | 160000001 | ApeKI | D4 | Euphrasia | spp | wildcollected |
| HGHVLBBXY | 3 | GATGTC | T330 | Euphrasia1 | D | 5 | 100000140 | 160000001 | ApeKI | D5 | Euphrasia | spp | wildcollected |
| HGHVLBBXY | 3 | TGTTAC | T331 | Euphrasia1 | D | 6 | 100000141 | 160000001 | ApeKI | D6 | Euphrasia | spp | wildcollected |
| HGHVLBBXY | 3 | CAGTTA | D332 | Euphrasia1 | D | 7 | 100000142 | 160000001 | ApeKI | D7 | Euphrasia | spp | wildcollected |
| HGHVLBBXY | 3 | GCCTAT | D333 | Euphrasia1 | D | 8 | 100000143 | 160000001 | ApeKI | D8 | Euphrasia | spp | wildcollected |
| HGHVLBBXY | 3 | AGTGGC | D334 | Euphrasia1 | D | 9 | 100000144 | 160000001 | ApeKI | D9 | Euphrasia | spp | wildcollected |
| HGHVLBBXY | 3 | TGACCT | T335 | Euphrasia1 | D | 10 | 100000145 | 160000001 | ApeKI | D10 | Euphrasia | spp | wildcollected |
| HGHVLBBXY | 3 | TTGCAC | T336 | Euphrasia1 | D | 11 | 100000146 | 160000001 | ApeKI | D11 | Euphrasia | spp | wildcollected |
| HGHVLBBXY | 3 | CTAGCT | D337 | Euphrasia1 | D | 12 | 100000147 | 160000001 | ApeKI | D12 | Euphrasia | spp | wildcollected |
| HGHVLBBXY | 3 | TAGTGC | D338 | Euphrasia1 | E | 1 | 100000148 | 160000001 | ApeKI | E1 | Euphrasia | spp | wildcollected |
| HGHVLBBXY | 3 | GTTCCA | D339 | Euphrasia1 | E | 2 | 100000149 | 160000001 | ApeKI | E2 | Euphrasia | spp | wildcollected |
| HGHVLBBXY | 3 | TGAATC | D340 | Euphrasia1 | E | 3 | 100000150 | 160000001 | ApeKI | E3 | Euphrasia | spp | wildcollected |
| HGHVLBBXY | 3 | CGCCAT | T341 | Euphrasia1 | E | 4 | 100000151 | 160000001 | ApeKI | E4 | Euphrasia | spp | wildcollected |
| HGHVLBBXY | 3 | ATCGTC | T342 | Euphrasia1 | E | 5 | 100000152 | 160000001 | ApeKI | E5 | Euphrasia | spp | wildcollected |
| HGHVLBBXY | 3 | GTCACT | D343 | Euphrasia1 | E | 6 | 100000153 | 160000001 | ApeKI | E6 | Euphrasia | spp | wildcollected |
| HGHVLBBXY | 3 | CATCGC | T344 | Euphrasia1 | E | 7 | 100000154 | 160000001 | ApeKI | E7 | Euphrasia | spp | wildcollected |
| HGHVLBBXY | 3 | AGGTCT | T345 | Euphrasia1 | E | 8 | 100000155 | 160000001 | ApeKI | E8 | Euphrasia | spp | wildcollected |
| HGHVLBBXY | 3 | CCTGCA | T346 | Euphrasia1 | E | 9 | 100000156 | 160000001 | ApeKI | E9 | Euphrasia | spp | wildcollected |
| HGHVLBBXY | 3 | GTACTC | D347 | Euphrasia1 | E | 10 | 100000157 | 160000001 | ApeKI | E10 | Euphrasia | spp | wildcollected |
| HGHVLBBXY | 3 | ACCTGC | D348 | Euphrasia1 | E | 11 | 100000158 | 160000001 | ApeKI | E11 | Euphrasia | spp | wildcollected |
| HGHVLBBXY | 3 | CTTGAC | T349 | Euphrasia1 | E | 12 | 100000159 | 160000001 | ApeKI | E12 | Euphrasia | spp | wildcollected |
| HGHVLBBXY | 3 | ACCGCT | T350 | Euphrasia1 | F | 1 | 100000160 | 160000001 | ApeKI | F1 | Euphrasia | spp | wildcollected |
| HGHVLBBXY | 3 | GCTCAC | D351 | Euphrasia1 | F | 2 | 100000161 | 160000001 | ApeKI | F2 | Euphrasia | spp | wildcollected |
| HGHVLBBXY | 3 | CTATGC | D352 | Euphrasia1 | F | 3 | 100000162 | 160000001 | ApeKI | F3 | Euphrasia | spp | wildcollected |
| HGHVLBBXY | 3 | CAGCCT | T353 | Euphrasia1 | F | 4 | 100000163 | 160000001 | ApeKI | F4 | Euphrasia | spp | wildcollected |
| HGHVLBBXY | 3 | GCCATC | T354 | Euphrasia1 | F | 5 | 100000164 | 160000001 | ApeKI | F5 | Euphrasia | spp | wildcollected |
| HGHVLBBXY | 3 | CGCTCA | D355 | Euphrasia1 | F | 6 | 100000165 | 160000001 | ApeKI | F6 | Euphrasia | spp | wildcollected |
| HGHVLBBXY | 3 | ACGCTC | D356 | Euphrasia1 | F | 7 | 100000166 | 160000001 | ApeKI | F7 | Euphrasia | spp | wildcollected |
| HGHVLBBXY | 3 | CTGATC | D357 | Euphrasia1 | F | 8 | 100000167 | 160000001 | ApeKI | F8 | Euphrasia | spp | wildcollected |
| HGHVLBBXY | 3 | CCAGTC | T358 | Euphrasia1 | F | 9 | 100000168 | 160000001 | ApeKI | F9 | Euphrasia | spp | wildcollected |
| HGHVLBBXY | 3 | TACGCC | T359 | Euphrasia1 | F | 10 | 100000169 | 160000001 | ApeKI | F10 | Euphrasia | spp | wildcollected |
| HGHVLBBXY | 3 | CCGTAC | D360 | Euphrasia1 | F | 11 | 100000170 | 160000001 | ApeKI | F11 | Euphrasia | spp | wildcollected |
| HGHVLBBXY | 3 | TCGACC | D361 | Euphrasia1 | F | 12 | 100000171 | 160000001 | ApeKI | F12 | Euphrasia | spp | wildcollected |
| HGHVLBBXY | 3 | GCATCC | D362 | Euphrasia1 | G | 1 | 100000172 | 160000001 | ApeKI | G1 | Euphrasia | spp | wildcollected |
| HGHVLBBXY | 3 | CGTACC | D363 | Euphrasia1 | G | 2 | 100000173 | 160000001 | ApeKI | G2 | Euphrasia | spp | wildcollected |
| HGHVLBBXY | 3 | ATTCAGT | D364 | Euphrasia1 | G | 3 | 100000174 | 160000001 | ApeKI | G3 | Euphrasia | spp | wildcollected |
| HGHVLBBXY | 3 | TATCTGA | T365 | Euphrasia1 | G | 4 | 100000175 | 160000001 | ApeKI | G4 | Euphrasia | spp | wildcollected |
| HGHVLBBXY | 3 | TTCAAGT | T366 | Euphrasia1 | G | 5 | 100000176 | 160000001 | ApeKI | G5 | Euphrasia | spp | wildcollected |
| HGHVLBBXY | 3 | GAGCAGT | D367 | Euphrasia1 | G | 6 | 100000177 | 160000001 | ApeKI | G6 | Euphrasia | spp | wildcollected |
| HGHVLBBXY | 3 | TCTAGGA | D368 | Euphrasia1 | G | 7 | 100000178 | 160000001 | ApeKI | G7 | Euphrasia | spp | wildcollected |
| HGHVLBBXY | 3 | ACACGGT | D369 | Euphrasia1 | G | 8 | 100000179 | 160000001 | ApeKI | G8 | Euphrasia | spp | wildcollected |
| HGHVLBBXY | 3 | GACGTGA | D370 | Euphrasia1 | G | 9 | 100000180 | 160000001 | ApeKI | G9 | Euphrasia | spp | wildcollected |
| HGHVLBBXY | 3 | TCATAGT | D371 | Euphrasia1 | G | 10 | 100000181 | 160000001 | ApeKI | G10 | Euphrasia | spp | wildcollected |
| HGHVLBBXY | 3 | TTACGAT | D372 | Euphrasia1 | G | 11 | 100000182 | 160000001 | ApeKI | G11 | Euphrasia | spp | wildcollected |
| HGHVLBBXY | 3 | GGCTAGA | D373 | Euphrasia1 | G | 12 | 100000183 | 160000001 | ApeKI | G12 | Euphrasia | spp | wildcollected |
| HGHVLBBXY | 3 | AATCGTT | D374 | Euphrasia1 | H | 1 | 100000184 | 160000001 | ApeKI | H1 | Euphrasia | spp | wildcollected |
| HGHVLBBXY | 3 | CTATGGA | D375 | Euphrasia1 | H | 2 | 100000185 | 160000001 | ApeKI | H2 | Euphrasia | spp | wildcollected |
| HGHVLBBXY | 3 | TACGGTA | D376 | Euphrasia1 | H | 3 | 100000186 | 160000001 | ApeKI | H3 | Euphrasia | spp | wildcollected |
| HGHVLBBXY | 3 | ACTATGT | D377 | Euphrasia1 | H | 4 | 100000187 | 160000001 | ApeKI | H4 | Euphrasia | spp | wildcollected |
| HGHVLBBXY | 3 | CGTGAAT | T378 | Euphrasia1 | H | 5 | 100000188 | 160000001 | ApeKI | H5 | Euphrasia | spp | wildcollected |
| HGHVLBBXY | 3 | TTGCAGA | D379 | Euphrasia1 | H | 6 | 100000189 | 160000001 | ApeKI | H6 | Euphrasia | spp | wildcollected |
| HGHVLBBXY | 3 | AACTTGT | D380 | Euphrasia1 | H | 7 | 100000190 | 160000001 | ApeKI | H7 | Euphrasia | spp | wildcollected |
| HGHVLBBXY | 3 | TGACGTA | D381 | Euphrasia1 | H | 8 | 100000191 | 160000001 | ApeKI | H8 | Euphrasia | spp | wildcollected |
| HGHVLBBXY | 3 | GCTATAA | D382 | Euphrasia1 | H | 9 | 100000192 | 160000001 | ApeKI | H9 | Euphrasia | spp | wildcollected |
| HGHVLBBXY | 3 | ATCGTAT | T383 | Euphrasia1 | H | 10 | 100000193 | 160000001 | ApeKI | H10 | Euphrasia | spp | wildcollected |
| HGHVLBBXY | 3 | TACTGAT | D384 | Euphrasia1 | H | 11 | 100000194 | 160000001 | ApeKI | H11 | Euphrasia | spp | wildcollected |
