# Supplementary material for: Is there hybridization between diploid and tetraploid Euphrasia in a secondary contact zone?
Source: Am J Bot. 2022 Dec 26;110(1):e16100. doi: 10.1002/ajb2.16100 (PMC10107515; doi:10.1002/ajb2.16100)
Supplement: Supplementary file 3 — Appendix S3. Hierarchical analysis of molecular variance (AMOVA) for ITS and 356 SNPs generated via GBS for diploid Euphrasia rostkoviana and tetraploid E. arctica. [file AJB2-110-0-s002.docx]

**Appendix S3.** Hierarchical Analysis of Molecular Variance (AMOVA) for ITS and 356 SNPs generated via GBS for diploid *Euphrasia rostkoviana* and tetraploid *E. arctica*. The total variation is partitioned between and within ploidy level. Degrees of freedom (df), the variance of each of the observations (Sum sq), and percentage of variation explained by each level of variation (% Var) are reported. The significance of the components of variance are reported as p-values.

| **Data set** | **Variation** | **DF** | **Sum Sq** | **% Var** | **p-Monte Carlo** |
| --- | --- | --- | --- | --- | --- |
| **A) ITS** | Between ploidy | 1 | 454.9 | 99.5 | 0.0001 |
|  | Within ploidy | 60 | 4.9 | 0.5 |  |
|  | Total | 61 | 459.8 | 100 |  |
| **B) GBS** | Between ploidy | 1 | 56 | 78.4 | 0.0001 |
|  | Within ploidy | 90 | 30.3 | 21.6 |  |
|  | Total | 91 | 86.3 | 100 |  |
