# Supplementary material for: Is there hybridization between diploid and tetraploid Euphrasia in a secondary contact zone?
Source: Am J Bot. 2022 Dec 26;110(1):e16100. doi: 10.1002/ajb2.16100 (PMC10107515; doi:10.1002/ajb2.16100)

**Appendix S4.** Distributions of parameters fitted to the models with constant gene flow (blue), secondary contact (green), and without gene flow (grey). See Figure 4A for schematics of the models with parameters shown. Models include effective population sizes (N_e_), and are either with or without (w/o) gene flow (GF) between diploids (dipl) and tetraploids (tet).


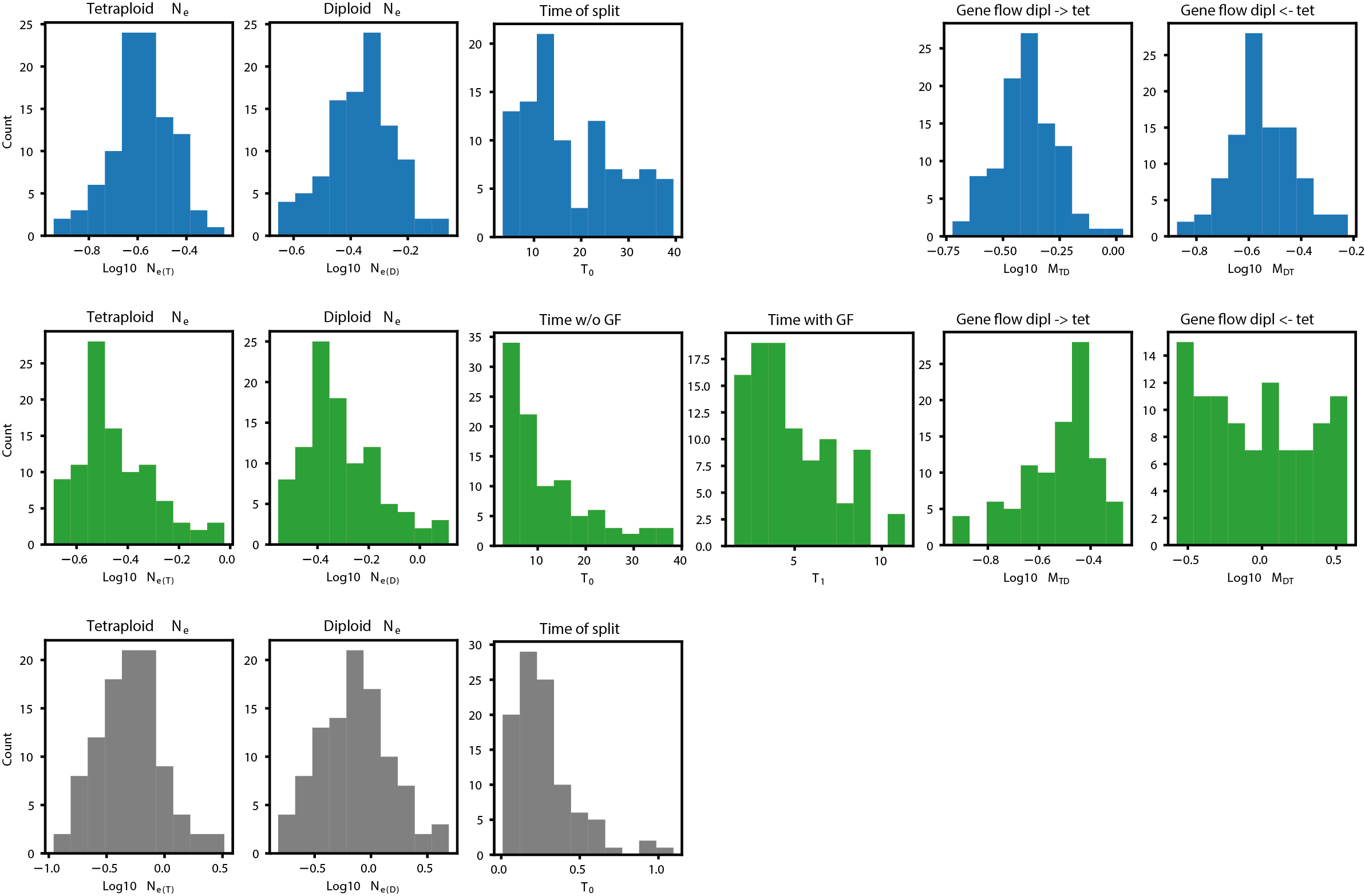

Supplement: Supplementary file 4 — Appendix S4. Distributions of parameters fitted to the models with constant gene flow, with secondary contact, and without gene flow. [file AJB2-110-0-s003.docx]
